# Asking the right questions: interrogating the logic and assumptions of paradigms used to investigate interactions between procedural and declarative memory in category learning

**DOI:** 10.3389/fcogn.2024.1505513

**Published:** 2025-01-07

**Authors:** Priya B. Kalra

**Affiliations:** Department of Psychology, Western Institute for Neuroscience, University of Western Ontario, London, ON, Canada

**Keywords:** declarative learning, procedural learning, category learning, process-dissociation procedure, multiple memory systems

## Abstract

In this mini-review, the methods used to investigate interactions between procedural and declarative systems in category learning are considered. Methods that were originally used to establish dissociations between memory systems may be biased toward demonstrating competition between them. In contrast, a modification of Jacoby's Process Dissociation Procedure allows researchers to consider the relative contributions of multiple processes involved in task completion. The original PDP was designed to consider the contributions of recall and familiarity to recognition, but the logic of the PDP can be applied to the contributions of procedural and declarative processes in category learning. Suggestions for improving the possibility of detecting cooperation between systems using the PDP are given.

At this point, the existence of multiple dissociable memory systems is widely accepted on the basis of converging evidence from neuropsychological, neuroimaging, and behavioral evidence (Cohen et al., 1985; Gabrieli, 1998; Squire, 2004; Zola-Morgan and Squire, 1993). As a field, we can now turn to question such as how these systems may or may not interact under normal circumstances (Ashby and Maddox, 2011; Freedberg et al., 2020). This review is concerned with the potential forms of interaction between two forms of memory in the context of human category learning: declarative and procedural. Declarative memory is understood to require certain medial temporal lobe (MTL) structures, including the hippocampus, to potentially take place quickly (such as one-shot learning), and to often yield verbalizable knowledge. While there are several forms of non-declarative memory, in this review I am focusing in particular on procedural memory, which is understood to require certain basal ganglia structures (especially the caudate nucleus in the dorsal striatum), to take place incrementally, and to yield actionable but non-verbalizable knowledge. While the differences in necessary neural structures established the distinctness of each system, given the other characteristics (e.g., slow vs. fast learning), we can infer that it is not only the implementational level at which these systems for learning differ, but more than likely the algorithmic and computational levels as well (Marr, 1982). In other words, not only do these systems have different neural substrates, but they are optimized for solving different kinds of problems and in different ways.

Given these differences, it may seem surprising that there are several domains of learning in which it appears that either declarative or procedural learning could be used. These include navigation (McDonald et al., 2004; McDonald and White, 1995; White and McDonald, 2002), learning sequences (Robertson, 2022; Song et al., 2007; Willingham et al., 2002; Willingham and Goedert-Eschmann, 1999; Witt and Willingham, 2006), and learning rules (Ullman, 2016). There have been several suggestions for how the two systems may interact in these situations. One possibility is that the two systems are simply redundant, encoding information in parallel, but not interacting. Such redundancy can be useful in cases where one system is impaired (for example by trauma to the brain), and the remaining system can compensate (Hartley and Burgess, 2005; Moody et al., 2004). There are also suggestions that the systems may compete during encoding, with some arbitrator in the brain declaring the more effective system the “winner.” However, there is also evidence from several real-world domains, including language use (Ullman, 2004, 2016), and skilled tool use (Roy and Park, 2010; Roy et al., 2015; Gregory et al., 2016), that the two systems may act cooperatively. For example, in a study of predictors of novel language learning, not only were both declarative and procedural learning ability predictive, but in fact an interaction between the two factors suggesting cooperation was also observed (Pili-Moss, 2022).

Category learning is another domain in which either procedural or declarative learning could be used: the encoding algorithm for each system could provide, if not the optimal solution, then at least a functional solution. However, in contrast to the examples of co-operative interaction above, the dominant model of interaction between procedural and declarative category learning is competition, either at encoding or retrieval/application (Freedberg et al., 2020; Poldrack and Packard, 2003). However, the dominance of this model may come from a case of “looking where the light is”: studies designed to highlight the separability of declarative and procedural memory systems would naturally tend away from demonstrating their interrelatedness. This may be an artifact of the struggle to establish the existence of multiple systems for category learning (that can be mapped to multiple memory systems) (Minda et al., 2024; Ashby and O'Brien, 2005). On the other hand, if we assume that both systems are contributing to categorization, then rather than asking “which system is being used,” we should ask “what is the contribution of each system [to categorization]”? This is the question that Jacoby (1991) posed for the roles of familiarity and recollection in recognition, and his solution, process dissociation, can be fruitfully applied to the contributions of declarative and procedural systems to category learning.

Briefly, the process-dissociation procedure first assumes that both processes contribute to performance of the task, rather than trying to establish the process purity of the task (which is inherently problematic: see Dunn and Kirsner, 1989; Reingold and Merikle, 1990). The task is then administered under facilitation and interference conditions. In the facilitation condition, both processes suggest the same response (Response = A + B). In the interference condition, each process suggests a different response (Response = A − B). The two conditions can then be compared to determine the contributions of each process to the task (solve a system of 2 equations to find two unknown variables) (Jacoby, 1991; Yonelinas and Jacoby, 2012). The process-dissociation procedure has been used beyond the original context to examine, for example, automatic and controlled contributions in perception and social psychology (Yonelinas and Jacoby, 2012); however, to our knowledge, the process-dissociation procedure has not been applied to examine the contribution of automatic (procedural) and controlled (declarative) processes in categorization.

In this review, we will not attempt to review the extensive literature on interaction between procedural and declarative memory (for an excellent comprehensive review, see Freedberg et al., 2020), but to specifically investigate the logic of the methods that have been used to probe interactions between procedural and declarative category learning, and to suggest where a process-dissociation perspective could be helpful. In this way, we contribute to integrating perspectives not only on declarative and procedural memory, but how a paradigm from one area of memory research can be applied to another, highlighting the need for dialogue within memory research.

## Types of category learning: deterministic and probabilistic

Category learning paradigms may be deterministic or probabilistic. In deterministic category learning, each stimulus is associated with only one category, and the given feedback is consistent regarding category membership. In contrast, in probabilistic category learning tasks (also known as probabilistic classification tasks aka PCT, such as the “Weather Prediction Task”), the association between each cue and the outcome (category) is probabilistic. Some cues are more consistent than others (for example, consistent = predicting outcome/category A 90% of the time and B 10% of the time; inconsistent = 55% outcome A, 45% outcome B). Thus, the same combination of cues may sometimes lead to one outcome (e.g., Category A) and sometimes the other (e.g., Category B; Knowlton et al., 1994). The probabilistic structure deters explicit hypothesis testing and produces lower rates of explicit knowledge inference by participants. Despite this difference in feedback consistency, probabilistic and deterministic category learning have similar task demands, and individual performance differences in these two tasks are correlated, suggesting shared processes or mechanisms (Kalra et al., 2019).

Optimal performance in a deterministic category learning paradigm could depend on either declarative or procedural learning mechanisms, depending on the configuration of stimulus space and placement of the category boundary. The stimulus space is often two-dimensional, such as the frequency and orientation of sine wave gratings. For simple, verbalizable category structures, such as those that require comparison along only one dimension (often referred to as “Rule-Based” categories or paradigms), declarative learning is efficient for reaching high levels of accuracy. In contrast, complex deterministic category structures that cannot be easily verbalized, such as those requiring integrating information across two dimensions simultaneously (“information-integration” category structures) are more effectively learned by an implicit, feedback-driven procedural approach Behavioral, neuropsychological, and neuroimaging evidence suggest that rule-based category learning depends on declarative processes, many mediated by medial temporal lobe structures (Ashby et al., 1998; Maddox et al., 2004; Waldron and Ashby, 2001; Zeithamova and Maddox, 2006), while information-integration category learning depends on procedural learning mechanisms that are mediated primarily by the striatum (Ashby and Ennis, 2006; Filoteo et al., 2005; Nomura et al., 2007).

In probabilistic category learning paradigms, cue combinations and cue-outcome association probabilities are generally too complex to be effectively learned through declarative methods (such as memorizing particular cue-outcome pairs). Like information-integration deterministic category learning, probabilistic category learning depends on procedural learning mechanisms that are mediated primarily by the striatum (Foerde and Shohamy, 2011b; Knowlton et al., 1994, 1996a,b; Shohamy et al., 2004; Squire et al., 1994).

## Methods of investigating interactions: logic, assumptions, and findings

### Manipulation of task demands

A common strategy in behavioral category learning studies has been manipulation of the stimulus space to favor one system over the other. As discussed above, this is the rationale for using unidimensional (rule-based) or bi/multi-dimensional (information integration) category boundaries in deterministic category learning. The optimal solution for unidimensional category structures is more quickly reached by the declarative system, but the declarative system is ill-suited to find the optimal solution for bi/multi-dimensional category boundaries.

However, it is impossible to guarantee that participants will in fact use the optimal strategy. As Jacoby (1991) cautioned, we must not overidentify processes with the tasks used to measure them, particularly because the tasks can rarely be process pure. This has been a problem for interpretation of probabilistic classification task (PCT) results. Despite the fact that procedural learning strategies are optimal in this task, normal control participants do attempt to learn declaratively at first, and by the end of training may have gained some declarative knowledge of cue-outcome associations (Gluck et al., 2002; Meeter et al., 2008; Shohamy et al., 2004).

One way to address this issue is *post-hoc* modeling of individual participant strategies, which has been used in probabilistic classification (e.g., Gluck et al., 2002; Knowlton et al., 1994; Meeter et al., 2006, 2008; Shohamy et al., 2004) and category learning (Ashby and Maddox, 1993; Maddox and Ashby, 1993). In this type of modeling, the idealized response pattern for each strategy is generated, then individual performance is compared to each of these ideals and labeled according to which ideal most closely matches the individual's actual performance. This kind of modeling can be used to confirm that participants are using the assumed system to or to identify participants who are not (for example, in II category learning, some participants tend to perseverate with ineffective unidimensional rules). However, note that the assumption of single-process contribution to response (within a trial or block) is still in place. This type of modeling does not include a way to discern contributions of dual (or multiple) processes within a trial.

### Lesion studies

The second method is selectively lesioning one system while sparing the other. This is only feasible in animal studies or rare cases of human tragedy, in which case the lesion may not be anatomically and functionally specific. Temporary inhibition of areas on the cortical surface can be induced with transcranial stimulation, but the key necessary structures for each system are unfortunately not on the cortical surface. If a task can be performed by patients with amnesia, but not by patients with diseases of the basal ganglia (Parkinson's Disease, PD and Huntington's Disease, HD), then we infer that the task can be carried out by the procedural system alone (and vice versa)—note that we cannot necessarily infer that this would be the case under normal circumstances (i.e., no lesion, both systems available). For example, probabilistic classification can be performed at normal-like levels by amnesia patients, but not PD and HD patients (Foerde and Shohamy, 2011b; Knowlton et al., 1994, 1996a; Shohamy et al., 2004). In the case of category learning, information-integration category learning has also followed this pattern (Filoteo et al., 2001a,b; Filoteo and Maddox, 2014). Some evidence for the opposite pattern (MTL-dependence) has been found for rule-based categories (Janowsky et al., 1989; Leng and Parkin, 1988; Filoteo et al., 2001a).

Note that the above inferences rest on the assumptions that the processes do not normally interact, and that the tasks are process pure. However, if we assume that under normal (non-lesion) circumstances, the two processes both contribute to categorization behavior, our interpretations might differ. For example, if the systems were tightly interdependent, then one might expect to find lower overall performance for a single “widowed” system. However, if the remaining system thrives in the absence of the other, then we might suspect that under normal (non-lesion) circumstances its activity is somehow suppressed/inhibited by the availability of the other system. There is also the possibility that the remaining system is unaffected by the inactivation of its counterpart, which would suggest that under normal circumstances they may not interact at all.

Interestingly, although the amnesic patients eventually reach normal-like performance on the PCT, they do show an initial deficit (in the first 50 training trials). According to the logic above, this suggests an interdependence. Poldrack et al. (2001) speculated that this might be due to formation of representations by the declarative system early in learning (“chunking”) that are then used to track distributional information by the procedural system. Similarly, PD patients sometimes struggle with rule-based category learning, although their declarative systems (at least early in disease progression) should be relatively intact (Filoteo et al., 2001b). One possible interpretation of this finding is an interdependent relationship between declarative and procedural learning, although in this case the computational mechanism is not clear.

### Selective behavioral obstruction: dual-task and delayed feedback conditions

Another way to shift the task demands to favor one system or another is to add a condition that selectively disadvantages one system. Dual-task learning conditions (learning while doing a simultaneous distracting task) are often used to obstruct declarative learning. Procedural learning depends on immediate feedback, so using delayed feedback (for example, 1-back reinforcement, Smith et al., 2018) has been a tactic to selectively hinder procedural learning (Foerde and Shohamy, 2011a; Maddox et al., 2003; Maddox and Ing, 2005; Smith et al., 2014). The logic of functional obstruction is analogous to that of selective lesioning: can the task be performed while one system is obstructed? How does the other system respond?

Comparing performance and neural activity under single- and dual-task conditions, Foerde et al. (2006) observed that PCT performance and striatal activity associated with PCT performance were not affected by a secondary task, while declarative cue-outcome knowledge suffered in the dual-task condition. This fact argues against several possible forms of interaction. If the two systems were competing for resources, then the total metabolic burden on declarative memory (due to the secondary task) might have been higher, diverting resources from procedural memory, in which case we would expect to see less striatal activity in the dual-task phase. On the other hand, if competition between systems resulted in inhibition of procedural learning by declarative learning, then we might have expected to see greater striatal activity because the procedural system is disinhibited when the declarative system is suppressed. Neither difference was observed, suggesting that, at least in this paradigm, neither competition for resources nor disinhibition of a previously suppressed system occurred. The authors suggest that competition may take place later, at retrieval or use, rather than at encoding.

In the original use of the process-dissociation procedure, divided attention (often using a simultaneous secondary task) was used as a manipulation to hinder the contribution of recollection (rather than familiarity) to recognition, much as it is in these cases. However, to our knowledge no intervention exists to “block” familiarity (automatic processes) (as delayed feedback can be used to block procedural learning), so in this case studies of interaction between procedural and declarative learning may have an advantage. To leverage this advantage, studies of category learning could compare performance during control (A+B), dual-task (A only), and/or delayed feedback (B only) conditions.

### Facilitation or interference: steps toward process dissociation

Another behavioral method to investigate interactions between memory systems is to overtly state the declarative solution to participants and observe whether this affects procedural learning. This is analogous to half of the process-dissociation procedure, facilitation. Several demonstrations using this method have suggested that the declarative information does not improve performance in sequence learning (Sanchez and Reber, 2013; Willingham and Goedert-Eschmann, 1999). To date, one study has used this approach with category learning, finding that declarative knowledge of the optimal strategy improved rule-based, but not information-integration, category learning (Rosedahl et al., 2021).

Crossley and colleagues took a step closer to process-dissociation by creating a stimulus space that would lead to different responses from each system (i.e., putting the processes in opposition to each other, creating an interference condition, the second part of the PDP). Process purity is not a concern if each systems suggests a different response. Crossley and colleagues (Ashby and Crossley, 2010; Crossley and Ashby, 2015; Turner et al., 2017) demonstrated that parallel encoding, leading to different solutions by the procedural and declarative systems, can take place, but that only the output of the declarative system was used during training. They paired this with a “behavioral knockout” of procedural learning using delayed feedback. When participants were tested on a (non-trained) section of the stimulus space for which only the procedural system could give the correct solution, those who were trained with delayed feedback were not able to do so, but those trained with immediate feedback were able to.^1^

A recent study has taken a further step toward using PDP in category learning by directly contrasting an interference condition with a facilitation condition. Kalra et al. (2024) created a stimulus space in which the items could be classified according to a complex but verbalizable rule based on the shapes of the items. Before training, we told participants this rule overtly, verbally, and with examples. However, the two categories differed probabilistically in the distribution of colors, with one category having more warm-colored items and the other having more cool-colored items. In the test phase, participants were asked to classify stimuli for which the shape and color information indicated the same category (facilitation condition) or in which they indicated opposite categories (interference condition). Reaction time was significantly slower in the interference condition than in the facilitation condition. We interpret these results as evidence that information from both systems may contribute even at a late stage (application/decision). Our results do not necessarily violate an assumption of information encapsulation (Fodor, 1983) or constitute “high-bandwidth leakage” (Robertson, 2022), but can be understood as two modules contributing to a decision-making or gate-keeping module. The gate-keeping module considers the contributions from both the declarative and procedural model, but may weight them differently.

### Neuroimaging

What about neuroimaging? Because the neuropsychology data established that the caudate nucleus is necessary for procedural learning and the hippocampus is necessary for declarative learning, the problem of reverse inference from neuroimaging data is somewhat ameliorated. We cannot infer that procedural learning is taking place based on caudate activity or that declarative learning is taking place based on MTL activity, but we can actually make a modus tollens inference: ~p →~q. That is, lack of caudate activity can be inferred as lack of procedural learning since the caudate is *necessary* for procedural learning, and lack of MTL activity can be inferred as lack of declarative learning since the MTL is *necessary* for declarative learning. However, conventional univariate fMRI analysis is based on contrasts in relative activity between conditions, which can lead to the appearance of lack of activity from one system.

More modern neuroimaging methods may be able to add clarity. Functional connectivity studies have suggested a cooperative relationship between procedural and declarative category learning (Albouy et al., 2008, 2013; Dickerson et al., 2011). To date, multivariate methods do not appear to have been used to examine contributions of declarative and procedural memory in category learning. Representational similarity analysis (Kriegeskorte et al., 2008) paired with a process-dissociation framework could be particularly informative. First, a stimulus space in which declarative and procedural processes can be put into either facilitative or interference conditions (as in Kalra et al., 2024) would be needed. Then, theoretical matrices based on the expected representations formed by procedural and declarative learning, respectively, could be constructed. Finally, the empirical multi-voxel patterns could be compared to each of the theoretical matrices, as well as the combination of the theoretical matrices using multiple regression. Fitted models could then reveal the relative contributions of each process to categorization behavior, which could vary across regions-of-interest.

## Conclusion

The relative lack of evidence for cooperation between procedural and declarative memory in category learning may reflect the fact that paradigms used to prove dissociation are sometimes used to make inferences about interactions. Adaptation of the process dissociation procedure to the study of procedural and declarative learning allows us to ask “what is the contribution of each system to performance” rather than assuming that tasks are process-pure. Such adaptation requires the creation of paradigms (such as stimulus spaces) in which procedural and declarative processes can be placed in opposition (interference) or agreement (facilitation). The study of interactions between procedural and declarative contributions has an advantage over the original recognition paradigm because procedural and declarative learning can each be separately obstructed, using delayed feedback or dual-task conditions, respectively. While traditional univariate fMRI analysis may be well-suited for establishing the separability of the systems, functional connectivity and representational similarity analysis (particularly when paired with a PDP-compatible stimulus space) may be better tools for examining their interaction.

## Footnotes

^1^This could be compared to the “unmasking” of previously learned place information in rats (McDonald et al., 2004; McDonald and White, 1995; White and McDonald, 2002).
